# Aurora Kinase Inhibitors: Current Status and Outlook

**DOI:** 10.3389/fonc.2015.00278

**Published:** 2015-12-21

**Authors:** Vassilios Bavetsias, Spiros Linardopoulos

**Affiliations:** ^1^Cancer Research UK Cancer Therapeutics Unit, Division of Cancer Therapeutics, The Institute of Cancer Research, London, UK; ^2^Breast Cancer Now, Division of Breast Cancer Research, The Institute of Cancer Research, London, UK

**Keywords:** aurora kinase, small molecules, hematologic diseases, kinase inhibitors, neuroblastoma

## Abstract

The Aurora kinase family comprises of cell cycle-regulated serine/threonine kinases important for mitosis. Their activity and protein expression are cell cycle regulated, peaking during mitosis to orchestrate important mitotic processes including centrosome maturation, chromosome alignment, chromosome segregation, and cytokinesis. In humans, the Aurora kinase family consists of three members; Aurora-A, Aurora-B, and Aurora-C, which each share a conserved C-terminal catalytic domain but differ in their sub-cellular localization, substrate specificity, and function during mitosis. In addition, Aurora-A and Aurora-B have been found to be overexpressed in a wide variety of human tumors. These observations led to a number of programs among academic and pharmaceutical organizations to discovering small molecule Aurora kinase inhibitors as anti-cancer drugs. This review will summarize the known Aurora kinase inhibitors currently in the clinic, and discuss the current and future directions.

## Introduction

**Figure 1:**
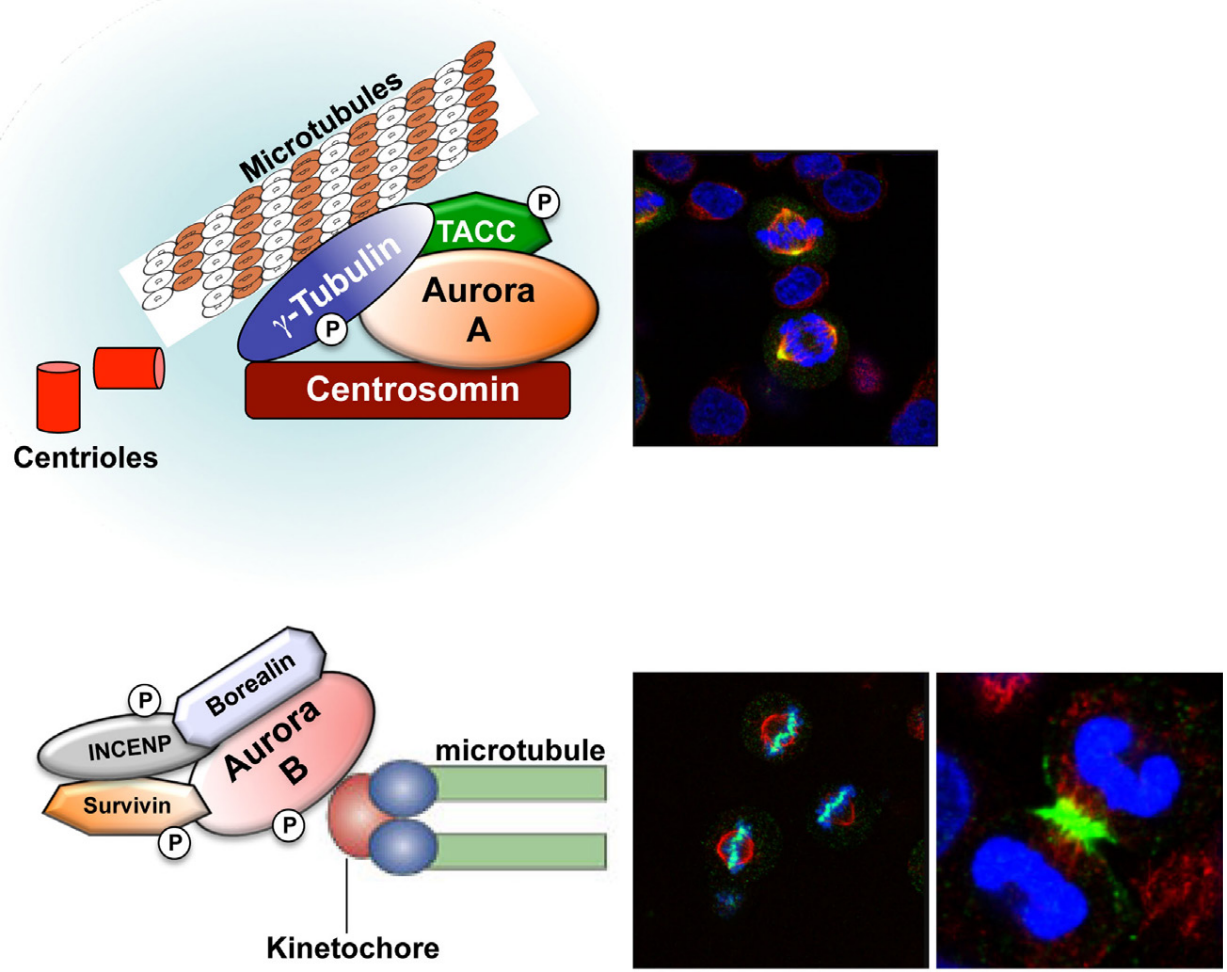
**Aurora-A and Aurora-B localization**. Aurora-A (yellow) localizes at the centrosome during interphase and at the mitotic poles and the adjacent spindle microtubules during mitosis. Aurora-B (green) localizes at the chromosomes in prophase, the centromere in prometaphase and metaphase, and the central mitotic spindle in anaphase.

The Aurora kinases are a family of highly conserved serine/threonine kinases that are important for faithful transition through mitosis (1–3). The gene for *Aurora-A*, maps to chromosome region 20q13.2, a region that has been found amplified in different human cancers. Aurora-A plays an important role in centrosome maturation, spindle assembly, meiotic maturation, and metaphase I spindle orientation [Figure 1 (2)]. Aurora-A function is regulated by degradation, phosphorylation, and dephosphorylation, with its kinase activity dependent upon phosphorylation of threonine 288 (Thr288) in the activation loop (2). Selective inhibition of Aurora-A results in inhibition of autophosphorylation of Aurora-A at Thr288, monopolar spindles, and G2-M arrest (4, 5). The *Aurora-B* gene maps to chromosome region 17p13.1 (1, 6) and this kinase forms part of the chromosomal passenger complex (CPC) with three non-enzymatic subunits: inner centromere protein (INCENP), Survivin, and Borealin [Figure 1 (7)]. The highly dynamic CPC is critical for chromosome condensation, chromosome orientation on the mitotic spindle, through correcting chromosome-microtubule attachment errors, and the spindle-assembly checkpoint (SAC), as well as the final stages of cytokinesis (8–11). Aurora-C expression has been reported in testis, thyroid, and placenta and in meiotically dividing gametes (2, 12–15). Recently, it was demonstrated that overexpression of Aurora-C induces abnormal cell division resulting in centrosome amplification and multinucleation in cells. NIH3T3 mouse fibroblasts overexpressing Aurora-C induced tumor formation in nude mice, demonstrating its oncogenic activity (16).

Aurora-A was first described in human cancer cell lines but has subsequently been found to be overexpressed in a broad range of human tumors, including primary colorectal carcinoma, gliomas, breast, ovarian, and pancreatic cancers (1, 17–20). Aurora-B is also overexpressed in human tumors such as gliomas, thyroid carcinoma, seminoma, and colon cancer (21–23).

**Figure 2:**
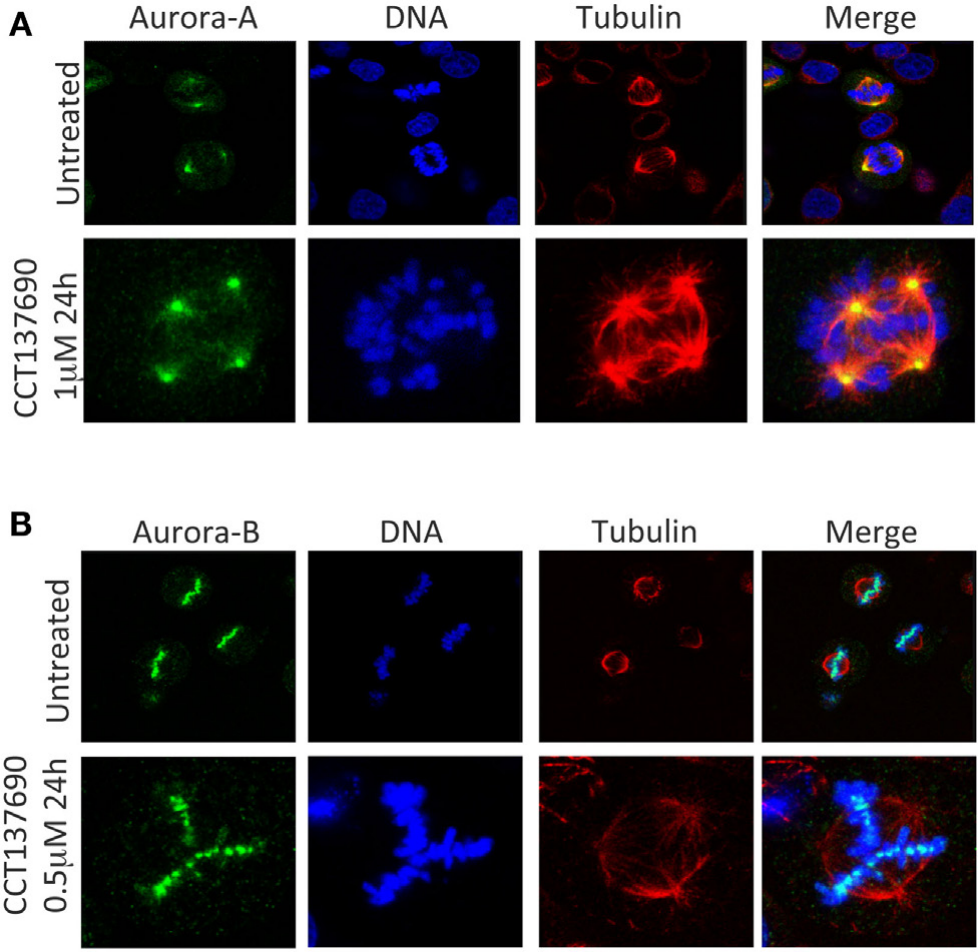
**Aurora-A and Aurora-B inhibition induces mitotic aberrations**. **(A)** Aurora-A inhibition induces temporarily a mitotic arrest, cells exit from mitosis with multipolar and monopolar spindle formation leading to apoptosis. **(B)** Aurora-B inhibition induces endoredublication, chromosome misalignments, and polyploidy.

The overexpression of the Aurora kinases and their association with genetic instability and aneuploidy in tumors suggests that a wide range of cancers could respond therapeutically to inhibitors of the Aurora kinases (24). Over the past decade, many pharmaceutical companies and academic institutions have reported the development of Aurora kinase inhibitors. Both Aurora-A and Aurora-B inhibitors induce cell death. However, they induce apoptosis through distinct mechanisms Figures 2A,B (25). Aurora-A inhibition induces defects in mitotic spindle assembly, which causes a transient spindle checkpoint-dependent mitotic arrest. This cell cycle arrest is not maintained, and subsequently, Aurora-A inhibited cells exit from mitosis leading to apoptosis, either by induction of a G1 arrest, followed by apoptosis, or by a p53-independent mechanism (25). In contrast, inhibition of Aurora-B also interferes with normal chromosome alignment during mitosis and overrides the mitotic spindle checkpoint causing polyploidy, failure of cytokinesis and endoreduplication followed by cell death at time more than 48 h (4, 5, 25). In a completely different direction, mechanistic studies have shown that Aurora-A inhibition also leads to MYCN degradation. The MYC family member MYCN, which is associated with amplification in the childhood tumor neuroblastoma, is stabilized by Aurora-A in a kinase-independent fashion, but involves a direct protein–protein interaction (26, 27). In this interaction, Aurora-A functions to sequester MYCN away from ubiquitin-mediated proteolytic degradation.

In this review, we will first present inhibitors of Aurora kinase family that have been recently assessed in clinical studies, with a focus on hematologic malignancies. Subsequently, we will discuss in more detail the areas in which inhibitors of Aurora kinases have shown greater promise, and we will highlight recent advances in the understanding of Aurora kinases biology that could be exploited with small-molecule modulators, bringing greater benefit to patients.

## Inhibitors of Aurora Kinases in Clinical Trials

### Barasertib (AZD1152)

Barasertib (AZD1152, Table 1) is a phosphate-based prodrug that is rapidly converted into barasertib-hQPA (Table 1) *in vivo* (28, 29). Barasertib-hQPA selectively inhibits the Aurora-B kinase (Aurora B-INCENP *K*i < 0.001 µM) over Aurora-A *K*i = 1.4 µM (28). In clinical studies, barasertib has been evaluated in patients with solid malignant tumors (30), advanced solid tumors (31), and hematologic cancers (32–35). The pharmacokinetic profiles of barasertib and barasertib-hQPA were investigated in a small phase I study in patients with newly diagnosed, relapsed or refractory acute myeloid leukemia AML (29), and in this study, one patient achieved a complete response. In a phase 1/2 study to investigate the safety, efficacy, and pharmacokinetics of barasertib in patients with advanced AML, barasertib was administered as a continuous 7-day infusion every 21 days and the MTD was established as 1200 mg (32). In part B of this study, 32 patients were treated with barasertib at 1200 mg. The most frequently observed grade ≥3 adverse events were febrile neutropenia and stomatitis/mucosal inflammation (32). In this investigation, treatment with barasertib resulted in an overall hematologic response rate of 25% with a manageable toxicity profile (32). A phase I study to investigate the safety, pharmacokinetics, and efficacy of barasertib in advanced AML was also undertaken in Japanese patients (33). Patients received barasertib as a continuous 7-day i.v. infusion every 21 days with no dose-limiting toxicities reported at doses up to and including 1200 mg, a dose that had been established as the MTD in a study by Lowenberg et al. (32). Febrile neutropenia, neutropenia, leukopenia, and thrombocytopenia were reported as the most frequent grade ≥3 adverse events (33), and in this investigation, an overall hematologic response rate of 19% was in line to response rates observed by Lowenberg et al. (32).

**Table 1:** **Aurora kinase inhibitors in clinical trials**.

Compound	Structure	Aurora inhibition	Reference
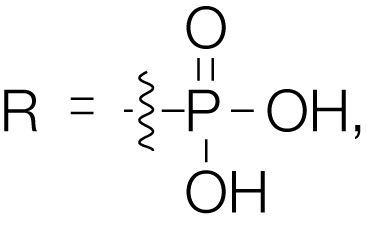 Barasertib (AZD1152) R = H, Barasertib-hQPA	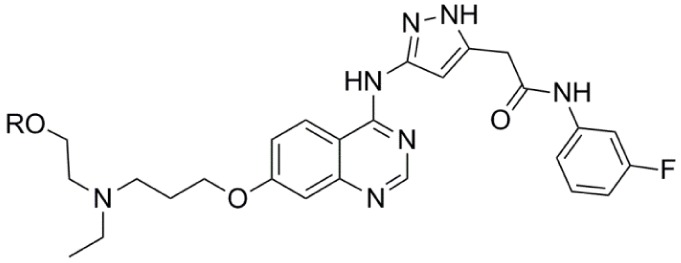	Aurora-A *K*i = 1.4 µM	(28–35)
		Aurora-B *K*i <0.001 µM; for barasertib-hQPA	
Alisertib (MLN8237)	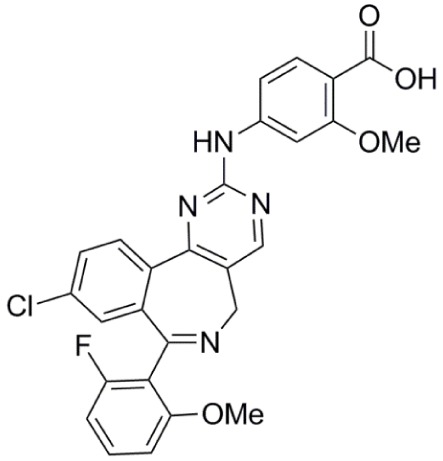	Aurora-A IC_50_ = 1.2 nM	(36–47)
		Aurora-B IC_50_ = 396.5 nM	
Danusertib (PHA-739358)	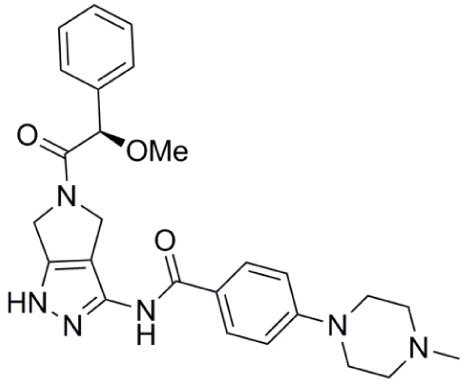	Aurora-A IC_50_ = 13 nM	(48–56)
		Aurora-B IC_50_ = 79 nM	
AT9283	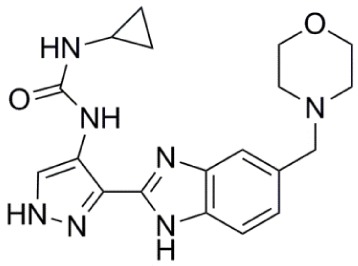	Aurora-A: 52% inhibition at 3 nM	(57–64)
		Aurora-B: 58% inhibition at 3 nM	
PF-03814735	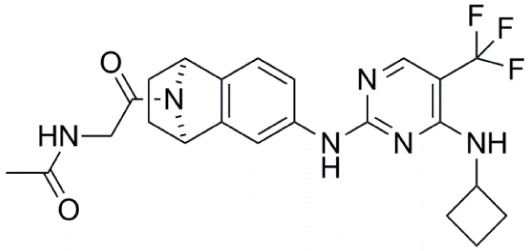	Aurora-A IC_50_ = 5 nM	(65, 66)
		Aurora-B IC_50_ = 0.8 nM	
AMG 900	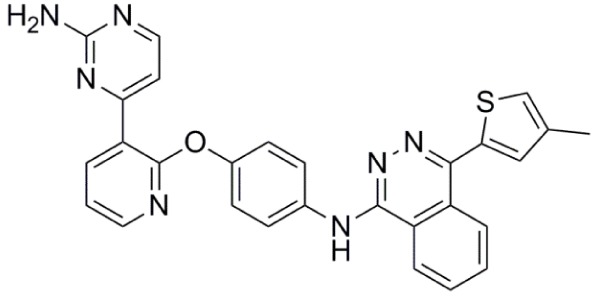	Aurora-A IC_50_ = 5 nM	(67–70)
		Aurora-B IC_50_ = 4 nM	

The safety and tolerability of barasertib has also been assessed in combination with low-dose cytosine arabinoside (LDAC) in elderly patients with newly diagnosed AML, considered unfit for intensive induction chemotherapy (34). In this study, patients received barasertib (7-day continuous intravenous infusion from day 1 to 7) and LDAC 20 mg (subcutaneously twice daily from day 1 to 10) of 28-day treatment cycles. This combination showed acceptable tolerability at doses up to and including 1000 mg of barasertib, which was established as the MTD; the most common grade ≥3 adverse event was febrile neutropenia. In this investigation, the combination of barasertib and LDAC resulted in an overall response rate of 45% (34).

The efficacy, safety and tolerability of barasertib *versus* LDAC in elderly patients with AML were also investigated in a randomized Phase II study (35). In this study, patients were randomized 2:1 to be administered either barasertib 1200 mg (as a 7-day continuous intravenous infusion) or LDAC 20 mg (subcutaneously twice daily for 10 days) in 28-days cycles. A higher objective complete response rate was reported with barasertib treatment: 35.4% compared with 11.5% observed with the LDAC treatment. The median overall survival for patients who received the barasertib treatment was longer relative to that observed with the LDAC treatment (35). Barasertib had a more toxic safety profile compared with that of LDAC treatment, the most commonly observed adverse events were stomatitis (71% in barasertib group versus 15% in LDAC group) and febrile neutropenia [67% in barasertib group versus 19% in LDAC group; (35)].

### Alisertib (MLN8237)

Alisertib (MLN8237; Table 1) is a selective inhibitor of the Aurora-A kinase, with an IC_50_ against Aurora-A of 1.2 and 396.5 nM against Aurora-B (36, 37). Alisertib has been extensively characterized using *in vitro* and *in vivo* preclinical models. It displays antiproliferative activity in a wide range of human tumor cell lines including lung, prostate, ovarian, and lymphoma cells (36). In relation to pediatric cancers, MLN8237 was active against neuroblastoma and Ewing sarcoma cell lines and efficacious in neuroblastoma and acute lymphoblastic leukemia (ALL) xenograft models (38). *In vitro*, alisertib also disrupts the viability of AML cell lines and primary AML cells (39) and significantly increases the *in vitro* and *in vivo* efficacy of nilotinib (40).

Given its broad spectrum of activity in preclinical models, alisertib has been evaluated in Phase I and II clinical trials in patients with advanced solid tumors (41–43), in children with refractory/recurrent solid tumors (44) and in hematologic malignancies. In a Phase I study of MLN8237 in relapsed/refractory multiple myeloma, Non-Hodgkin lymphoma, and chronic lymphocytic leukemia, patients received alisertib orally as either powder-in-capsule (PIC) or enteric-coated tablet (ECT) formulation (45). The most commonly reported grade ≥3 adverse events were neutropenia, thrombocytopenia, anemia, and leukopenia. In this study, partial responses were observed in 6 patients (13%), and 13 patients (28%) had stable disease for 1.9–11 months (45). The recommended phase II dose of alisertib (ECT formulation) was 50 mg twice daily for 7 days in 21-day cycles [i.e., a 7-day treatment followed by a 14-day recovery period; (45)].

Alisertib was evaluated in a Phase II study in patients with relapsed and refractory aggressive B- and T-Cell Non-Hodgkin lymphomas, and in this investigation, alisertib was administered orally at 50 mg, twice daily for 7 days in 21-day cycles (46). The most frequently observed grade ≥3 adverse events were neutropenia, leukopenia, anemia, and thrombocytopenia. In this study, the overall response rate for all treated patients was 27% (46). In an exploratory Phase II study, alisertib was assessed in AML and myelodysplastic syndrome [MDS; (47)]. In this investigation, alisertib was administered at 50 mg twice daily for 7 days in 21-day cycles and shown a modest single-agent antileukemic activity. In AML patients, a 17% overall response rate was observed with an additional seventeen patients (49%) having stable disease (47), but no responses were reported in MDS patients. The most commonly observed grade ≥3 adverse events were febrile neutropenia, anemia, thrombocytopenia, neutropenia, and fatigue (47).

### Danusertib (PHA-739358)

Danusertib (PHA-739358, Table 1) is a potent inhibitor of all three Aurora kinase isoforms (Aurora-A IC_50_ = 13 nM, Aurora-B IC_50_ = 79 nM, Aurora-C IC_50_ = 61 nM) (48, 49). In addition to the Aurora kinases, PHA-739358 has inhibitory activity against a number of other kinases with relevance as anticancer targets such as ABL, RET, and TRK-A (48, 49). PHA-739358 inhibits both the wild-type and mutant ABL kinase isoforms with clinical relevance, in particular ABL (T315I) which is one of the most common mutations found in imatinib-resistant patients (50).

In the clinical setting, danusertib has been assessed in patients with advanced or metastatic solid tumors (51, 52). In these Phase I and II studies, danusertib was generally well tolerated, neutropenia being one of the most commonly observed hematologic toxicities, but showed only marginal antitumor activity in patients with common advanced solid tumors who had failed systemic therapy (52). In addition, danusertib showed only minimal efficacy when it was assessed in a randomized Phase II study in patients with metastatic castration-resistant prostate cancer after docetaxel failure (53). In another Phase I study, danusertib was administered as 24-h infusion every 14 days with and without granulocyte colony-stimulating factor (G-CSF) and assessed in patients with advanced solid tumors (54). PHA-739358 has also been investigated in hematologic malignancies, including a phase II study in patients with chronic myeloid leukemia (CML) relapsing on imatinib or other targeted therapies (55). In this study, two complete hematologic responses were reported in patients that both carrying the T315I BCR–ABL mutation. Patients received danusertib by a once-weekly 6-h infusion, which was reportedly well tolerated (55). Danusertib was also assessed in phase I study in adult patients with accelerated or blastic phase CML and philadelphia chromosome-positive ALL resistant or intolerant to imatinib and/or other second generation ABL kinase inhibitors (56). In this investigation, danusertib was administered by 3-h IV infusion, daily for either 7 consecutive days in a 14-day cycle (schedule A) or 14 consecutive days in a 21-day cycle (schedule B). The most frequently reported grade 3–4 adverse events were anemia, diarrhea, and febrile neutropenia. Four (20%) of the twenty evaluable patients responded to treatment, all on schedule A treatment and carrying the T315I BCR-ABL mutation (56). Overall in this study, danusertib has shown an acceptable toxicity profile and also promising activity in patients with advanced hematologic malignancies resistant to imatinib and/or other second generation ABL kinase inhibitors (56).

### AT9283

AT9283 (Table 1) is a small-molecule multitargeted kinase inhibitor with potent Aurora kinase activity [Aurora-A: 52% inhibition at 3 nM; Aurora-B: 58% inhibition at 3 nM; (57)]. Other kinases inhibited by AT9283, with relevance as anticancer targets include JAK2, FLT-3, and ABL(T315I) (57). AT9283 showed potent antiproliferative activity against imatinib-resistant BCR-ABL^+^ cells including those carrying the ABL(T315I) mutation (58). AT9283 displayed potent inhibitory activity against a range of human solid tumor cell lines (59), and antiproliferative activity against a panel of human aggressive B-(non-Hodgkin lymphoma) B-NHL cell lines (60).

AT9283 was assessed in Phase I clinical trials in patients with advanced solid malignancies (61), advanced malignancies [administered as a weekly 24-h infusion; (62)] and in children and adolescents with solid tumors (63). A Phase I and pharmacodynamic study of AT9283 in patients with relapsed/refractory leukemia or myelofibrosis was also reported (64). In the initial part of this study, AT9283 was administered as a 72-h continuous infusion every 21 days; and in the second part of the investigation, infusion duration was increased sequentially (in 24-h increments) to 96 and 120 h. The MTD for a 72-h infusion was established as 108 mg/m^2^/d, and dose-limiting toxicities included myocardial infarction, hypertension, cardiomyopathy, tumor lysis syndrome, and pneumonia (64). In approximately one-third of patients with relapsed/refractory AML, bone marrow blasts decreased by at least 38% after treatment. However, these reductions in blasts were transient and no objective responses were achieved (64).

### PF-03814735

PF-03814735 (Table 1) is a potent, orally bioavailable inhibitor of both Aurora-A and Aurora-B kinases (Aurora-A IC_50_ = 5 nM, Aurora-B IC_50_ = 0.8 nM); and also inhibits several other kinases (e.g., FLT3, JAK2, TrkB, RET, MST3) by ≥90% at a compound concentration of 100 nM (65). PF-03814735 exhibited antiproliferative activity against a range of human tumor cell lines such as HCT-116, HL-60, A549, and H125 (65). In a Phase I clinical study, PF-03814735 was assessed in patients with advanced solid tumors (66). PF-03814735 was administered orally, once daily, as a single agent dosing at days 1–5 or 1–10 of 21-day cycles. Dose limiting toxicities included febrile neutropenia and increased levels of aspartate amino transferase. It was generally well tolerated with a clinically manageable adverse events profile but limited antitumor activity was reported with 19 patients achieving stable disease (66).

### AMG 900

AMG 900 (Table 1) is an orally bioavailable, potent and selective pan-Aurora kinase inhibitor (67–70). It inhibits Aurora-A, -B, and -C with IC_50_ values of 5, 4, and 1 nM respectively, and in cells shows a phenotype consistent with Aurora-B inhibition (67). AMG 900 displayed potent antiproliferative activity against a range of human tumor cells including cells lines resistant to paclitaxel and the Aurora kinase inhibitors AZD1152, MK-0457, and PHA739358 (67). Notably, AMG 900 was consistently potent against tumor cells irrespective of P-gp or BCRP status (67). Consistent with the *in vitro* findings, AMG 900 inhibited the growth of multiple human tumor xenograft models [e.g., MDA-MB-231, HCT116, NCI-H460-PTX (MDR), MES-SA, MES-SA-Dx5 (MDR)] *in vivo*, using either intermittent or continuous dosing schedules (67). The activity of AMG 900 as a single agent and in combination with paclitaxel or ixabepilone in multidrug-resistant TNBC (triple negative breast cancer) cell lines was also investigated (70). AMG 900 potently inhibited the growth of P-gp-expressing TNBC cell lines. In combination with paclitaxel or ixabepilone, AMG 900 enhanced the antiproliferative activity of these microtubule-targeting agents in TNBC cells *in vitro* and in human tumor xenograft models *in vivo* (70). AMG 900 is currently being evaluated in Phase I clinical trials in adult patients with advanced solid and hematologic cancers (68).

### Aurora Kinase Inhibitors in Hematologic Malignancies

**Figure 3:**
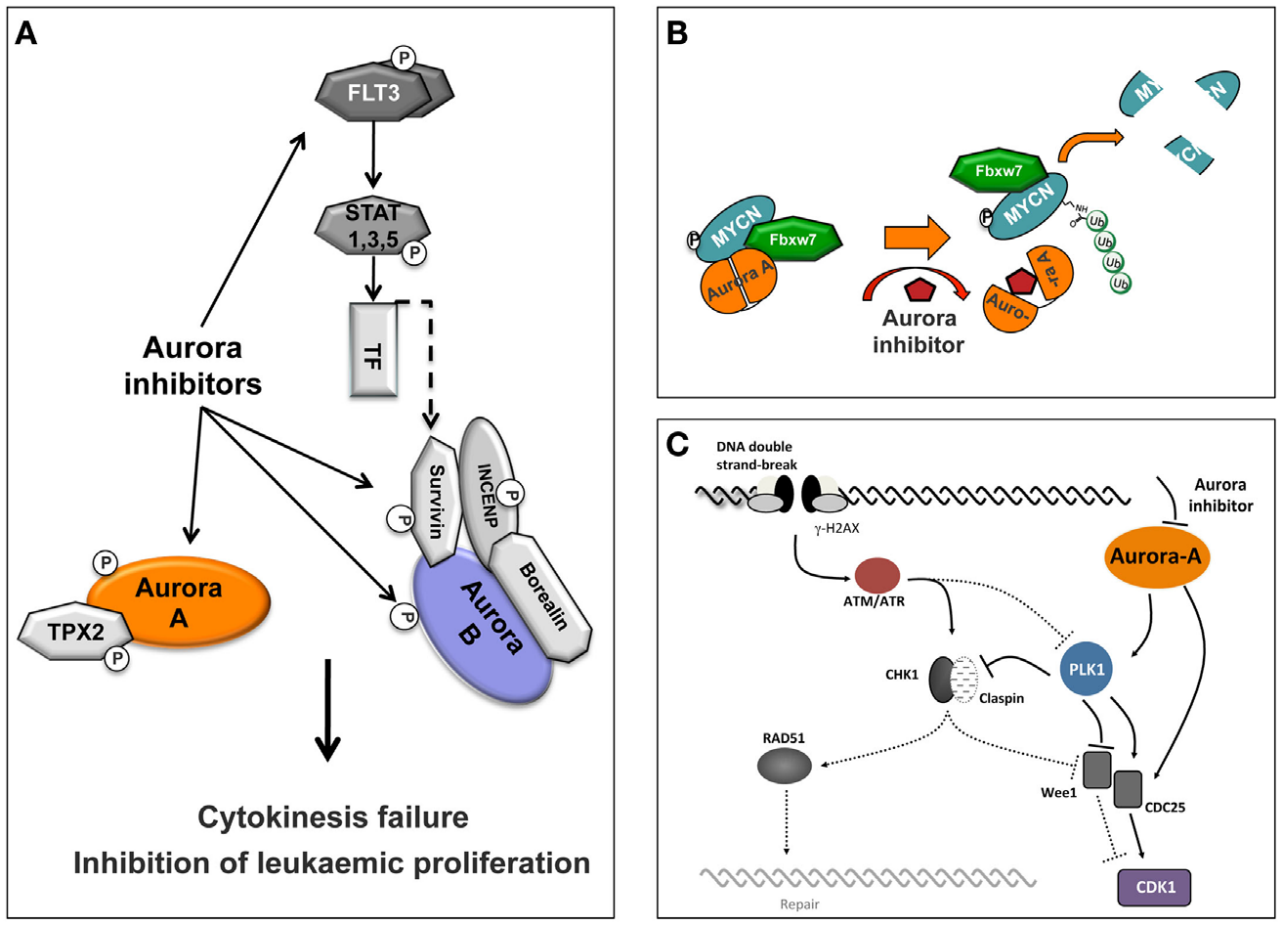
**Schematic representation of Aurora inhibitor-targeted pathways**. **(A)** Selective inhibition of Aurora-A or Aurora-B or dual specificity Aurora/FLT3 compounds induces cytokinesis failure and inhibition of leukemic proliferation. **(B)** Conformational changes of Aurora-A by a compound induce Aurora-A-FBXW7-MYCN complex disassembly and proteolytic degradation of MYCN. **(C)** Aurora-A is involved in DNA repair pathways and Aurora-A inhibitors confer increased cellular sensitivity to cell death.

The most profound explanation for the limited response of the Aurora kinase inhibitor’s in solid tumors in a clinical setting is possibly the need for drug exposures through a number of cell cycles (for the Aurora-B and pan Aurora inhibitors) or for a prolonged time in mitosis (for the Aurora-A inhibitors), to induce their maximum effect in tumor cells before severe toxic effects such as neutropenia appear. The clinical evaluation of Aurora kinase inhibitors, as discussed earlier, indicated toxicities that are consistent with the mechanism of action for this class of compounds. In clinical setting, efficacy against solid tumors is limited, but overall, Aurora kinase inhibitors showed greater promise against hematologic malignancies. The observed significant response in patients with hematologic cancers may be associated with their proliferation rate as well as with secondary pharmacology exhibited by some of these inhibitors which is related to the disease (Figure 3A).

AML is a heterogeneous class of leukemia, with prognosis predicted by a number of genetic and molecular abnormalities. Mutations of the fms-like tyrosine kinase 3 (*FLT3*) gene are one of the best characterized genetic alterations, which is frequently mutated in AML. These mutations can consist of internal tandem duplication (ITD) of the juxtamembrane domain coding region, or point mutations of the tyrosine kinase domain (TKD). Both FLT3-ITD and FLT3-TKD mutations result in ligand-independent proliferation and are associated with a poor prognosis in adults and children (71).

AZD1152 was the first selective Aurora-B inhibitor that was evaluated in pre-clinical and clinical studies. AZD1152 has demonstrated impressive efficacy in animal models using human tumor xenografts of AML cell lines with wild type and mutant FLT3 (72–74). Aurora-B inhibition was demonstrated, using phosphorylation of histone H3 (a known substrate of Aurora-B), as a biomarker for target engagement *in vitro* and *in vivo*. Polyploidy was the predominant phenotype associated with Aurora-B inhibition due to cytokinesis failure and endoreduplication. It has been also shown that a secondary target of AZD1152 is FLT3, resulting in higher sensitivity of FLT3-mutated cell lines MV4-11 and MOLM-13 to AZD1152 inhibition, compared to FLT3-WT cell lines (75). Based on these data, AZD1152 entered a number of clinical trials in patients with solid tumors or hematologic malignancies, including AML, as described previously in this review.

Although FLT3 kinase has attracted a great interest in recent years as a target for AML treatment, the clinical impact of early FLT3 inhibitors has been limited when used as single agents, due to acquired resistance (76). Newer FLT3 inhibitors with improved selectivity, pharmacokinetic and pharmacodynamic properties may have improved single-agent efficacy (77), but clinical resistance, including acquired secondary mutations in the FLT3-TKD, is emerging. However, FLT3 inhibitors exhibiting secondary kinase inhibition pharmacology showed promise in overcoming this resistance. For example, it was demonstrated that a human FLT3-ITD+ AML cell line harboring a secondary D835Y mutation, has high relative resistance to the FLT3 inhibitors AC220, MLN518, and Sorafenib, but not to CCT137690, a dual FLT3-Aurora kinase inhibitor (78). CCT241736, an advanced analog of CCT137690, is a preclinical development candidate for the treatment of human malignancies, and in particular AML in adults and children (79). CCT241736 is an orally bioavailable dual FLT3/Aurora kinase inhibitor that also inhibits clinically relevant FLT3-resistant mutants including FLT3-ITD and FLT3 [D835Y; (79)]. CCT241736 significantly inhibited the growth of MV4-11 human FLT3-ITD positive AML tumor xenografts *in vivo*, with biomarker modulation and free drug exposure consistent with dual FLT3 and Aurora kinase target inhibition (79).

### Aurora Inhibitors in Neuroblastoma

Neuroblastoma is the most common extra-cranial solid tumor of childhood, accounting for approximately 10% of pediatric tumors, which affects more than ten thousand children world-wide each year. Stage 4 neuroblastoma represents approximately 50% of cases with metastatic dissemination at diagnosis and its prognosis is poor. Therefore, novel therapeutic strategies are urgently needed to improve the prognosis of neuroblastoma patients. Amplification of the MYCN gene is associated with an aggressive form of neuroblastoma that results in a particularly poor clinical outcome (80). Knockout of MYCN protein by targeting with siRNA, or alternatively, destabilizing the protein using an inhibitor of the upstream PI3K signaling pathway, has been shown as an effective preclinical therapy for neuroblastoma (81, 82).

Initially, a study using the pan Aurora inhibitor CCT137690 showed that treatment of MYCN-amplified neuroblastoma cell lines inhibits cell proliferation and decreases MYCN protein expression (83). Importantly, in a transgenic mouse model of neuroblastoma (TH-MYCN) that overexpresses MYCN protein and is predisposed to spontaneous neuroblastoma formation, this compound significantly inhibits tumor growth (83). Later, an additional mechanistic study shown that Aurora-A forms a complex with MYCN in MYCN-amplified neuroblastoma cells, which protects MYCN from proteasomal degradation in mitosis (26). This activity was specific for Aurora-A, since neither Aurora-B inhibition nor depletion affected MYCN protein levels. Interestingly, stabilization of MYCN does not require the catalytic activity of Aurora-A (26). Furthermore, MYCN destabilization was not due to cell cycle arrest in G2/M due to inhibition of the catalytic activity of Aurora-A. However, crystallographic evidence showed that the Aurora-A-specific inhibitor MLN8054 (84) induces a DFG-up conformation, disrupting the Aurora-A/MYCN complex leading to MYCN degradation. The conclusions from this study were that disruption of the Aurora-A/MYCN complex promotes degradation of MYCN, mediated by the FBXW7 ubiquitin ligase. Therefore, inhibition of the Aurora kinases may be an effective strategy to treat MYCN-amplified neuroblastoma. In a completely different approach, a recent study provided evidence for an Aurora-A conformation-specific effect on proteolytic degradation of MYCN. CD532, an ATP-mimetic ligand, binds Aurora-A in a DFG-in, inactive conformation, which results in blocking both the kinase-dependent and independent functions of Aurora-A (27). Importantly, CD532 inhibits Aurora-A at low nanomolar concentrations and, in parallel, effects the proteolytic degradation of MYCN proposing an additional strategy to block MYCN in cancer (Figure 3B).

### Aurora Inhibitors and DNA Damage

A great effort has been focused on investigating different approaches to enhance the effect of Aurora kinase inhibitors in preclinical models and in clinical trials, including investigating the role of Aurora-A in DNA-damage response (DDR). It is known that Aurora-A activity is tightly regulated during the response to genotoxic agents and is important for a normal DDR (85, 86). There is an intricate connection between the DDR and the cell cycle at multiple levels. Once the homologous recombination (HR) machinery is fully active, the cell cycle is normally stalled by the activation of the DNA damage checkpoints. For the G2/M DNA damage checkpoint, the cell cycle arrest is mostly achieved by the regulation of CDC25 phosphatases and WEE1, either by the checkpoint kinases CHK1 and CHK2, or by the ATM/ATR kinases-dependent phosphorylation of PLK1 (Figure 3C). These two pathways converge to maintain an efficient inhibition of CDK1 and hence prevent cell cycle progression. Finally, during recovery from the DNA damage checkpoint, the signal emanating from the mitotic kinase PLK1 becomes dominant and stimulates cell cycle progression. Interestingly, during this late phase of the DDR, but also during unperturbed cell cycle, Aurora-A has been identified as the upstream activator of PLK1 (87, 88).

A recent study has shown that Aurora-A modulates the repair of DNA double-strand breaks [DSBs; (89)]. Aurora-A expression inhibits RAD51 recruitment to DNA DSBs, decreases DSB repair by HR and sensitizes cancer cells to PARP inhibition (89). This impairment of RAD51 function requires inhibition of CHK1 by PLK1. These results identify a novel function of Aurora-A in modulating the response to DNA DSB that likely contributes to carcinogenesis and suggest a novel therapeutic approach to the treatment of cancers overexpressing this protein. The connection of DDR with Aurora kinases triggered a number of trials combining radiotherapy with Aurora kinase inhibitors. AZD1152, the Aurora-B kinase inhibitor, was shown to enhance the effect of ionizing radiation (IR) in three different settings: neoadjuvant (AZD1152 before IR), adjuvant (IR before AZD1152), or concomitant treatments [AZD1152 plus one single IR dose; (90)]. A more pronounced tumor growth delay was observed in the neoadjuvant and adjuvant schedules as compared to the concomitant schedule. However, AZD1152 enhanced the efficacy of IR when concomitant IR was fractionated over several days. Histopathological examination revealed that AZD1152 + IR induced polyploidy, multinucleation, and micronuclei *in vivo*. Caspase inhibition or removal of the pro-apoptotic protein BAX did not ameliorate the long-term cell survival of AZD1152-treated cancer cells. In contrast, a chemical inhibitor of CHK1, CHIR124, sensitized cancer cells to the lethal effect of AZD1152, supporting the contention that AZD1152 mediates radiosensitization *in vivo* by enhancing mitotic catastrophe (90). More recent studies using the Aurora-A selective inhibitor MLN8237 showed the effect of MLN8237 with and without temozolomide or IR, on the proliferation of glioblastoma tumor stem-like cells. It was reported that Aurora-A inhibition by MLN8237 was synergistic with temozolomide and potentiated the effects of IR on colony formation in neurosphere glioblastoma tumor stem-like cells, supporting the potential of Aurora-A inhibitors as primary chemotherapy agents or biologic response modifiers in glioblastoma patients (91).

## Conclusion and Future Directions

Aurora kinase inhibitors were initially aimed to target solid tumors including ovarian, breast, lung and colon. Despite the wide use of different chemical classes of Aurora kinase inhibitors in clinical trials, a limited efficacy against solid tumors was observed. The most plausible explanation for this result may relate to the proliferation rate of cells in solid tumors being relatively slow. By the time that an Aurora kinase inhibitor had an effect through several cell cycles and mitoses in tumors, the faster proliferating bone marrow cells were severely affected. A break in treatment, to allow the patient to recover, enabled the tumors to continue growing. Regarding compound preclinical evaluation, it appears that for cell cycle inhibitors, particularly selective mitotic inhibitors, the existing pre-clinical efficacy models in which these compounds were evaluated are not predictive of the efficacy outcome in clinical trials. The disappointing outcome against solid tumors in the clinical setting led to a change in strategy with Aurora kinase inhibitors being evaluated in clinical trials against hematologic malignancies due to their higher homogeneity and higher proliferation rates relative to solid tumors. Higher response rates against hematologic malignancies were observed, in particular when the Aurora inhibitor exhibited a secondary anticancer pharmacology; for example, the inhibition of another oncogenic driver of hematologic cancers such as activated FLT3 in AML. In conclusion, simultaneous inhibition of an activated oncogene driver and Aurora kinases using compounds with dual pharmacology or selective Aurora inhibitors in combination with a selective oncogene driver inhibitor may be a strategy to achieve a significantly improved clinical outcome, and also overcome resistance.

## Author Note

Spiros Linardopoulos joined The Institute of Cancer Research (ICR) in September 2000 and hold an appointment as Team Leader in both the Breakthrough Breast Cancer Research Centre and the Cancer Research UK, Cancer Therapeutics Unit. Prior to the ICR, he worked at Onyx Pharmaceuticals, Richmond, CA, USA (1997–2000) as a Scientist. Before his move to the USA, he was awarded a Marie Curie Research Fellowship from the European Community as a postdoctoral researcher at the Beatson Institute for Cancer Research, Glasgow, UK (1993–1997). He pursued his graduate studies at the National Hellenic Research Foundation and obtained his PhD in Cellular and Molecular Biology from the University of Athens, Greece (1989–1993).

## Author Contributions

Dr. VB wrote the “Inhibitors of Aurora kinases in clinical trials” part of the Review. Dr. SL wrote the introduction, the use of Aurora kinase inhibitors in different human cancers, and the conclusions and future direction.

## Conflict of Interest Statement

The authors are employees of The Institute of Cancer Research, which has a commercial interest in Aurora inhibitors and operates a reward to inventors scheme.
